# From genes towards products and the significance of gene delivery

**DOI:** 10.1186/1753-6561-5-S7-I17

**Published:** 2011-09-13

**Authors:** Magnus Hertzberg

**Affiliations:** 1 SweTree Technologies AB

## 

SweTree Technologies is a plant and forest biotechnology company providing products and technologies to improve the productivity and performance properties of plants, wood and fiber for forestry and fiber related industries.

In the area of Biotech Trees, we have been testing and developing genes and biotech trees. Since 2002 we have successfully screened well over one thousand genes in our gene testing program. The program has been mainly directed towards increased growth, improved wood properties and stress tolerance. The current outcome of this program will be presented. The primary gene developing program is performed in hybrid aspen for which we have a very efficient transformation protocol. However for practical tree biotechnology the genes and DNA constructs have to be transformed into the best commercial clones. Therefore we have been setting up efficient transformation protocols for both *Eucalyptus* and *Populus* elite clones. Another crucial part of a practical tree biotechnology program is to test the GM trees with commercially interesting genes under field conditions. We have initiated field trials and plan to have over 35 different types of transgenic trees in field trials by the end of this year. These field experiments are performed by SweTree or in collaboration with academic partners. The importance and requirements for elite clone transformation and field trials will be discussed in the presentation.

